# Behavioural intervention to promote the uptake of planned care in urgent dental care attenders: study protocol for the RETURN randomised controlled trial

**DOI:** 10.1186/s13063-022-06418-2

**Published:** 2022-06-07

**Authors:** R. Harris, V. Lowers, C. Hulme, G. Burnside, A. Best, J. E. Clarkson, R. Cooke, M. Van Der Zande, R. Maitland

**Affiliations:** 1 Department of Public Health, Policy and Systems, Institute of Population Health, University of Liverpool, Whelan Building, Liverpool, L69 3GL UK; 2 Department of Public Health, Policy and Systems, Institute of Population Health, University of Liverpool, Liverpool, UK; 3 Health Economics Group, Institute of Health Research University of Exeter Medical School, Exeter, UK; 4 Department of Health Data Science, Institute of Population Health, University of Liverpool, Liverpool, UK; 5 Liverpool Clinical Trials Centre, Clinical Directorate, University of Liverpool, Liverpool, UK; 6 Division of Oral Health Sciences, Dental Hospital & School, Park Place, Dundee, UK; 7 School of Health, Science and Wellbeing, Staffordshire University, Stoke-on-Trent, ST4 2DE UK

**Keywords:** Randomised controlled trial, Inequalities, Dental, Attendance, Urgent care, Behavioural intervention, Primary care

## Abstract

**Background:**

People with disadvantaged backgrounds are less likely to visit the dentist for planned care, even though they have disproportionately poorer oral health. They are correspondingly more likely to experience dental problems and use urgent dental care, general practices and Accident and Emergency departments, which not only makes meeting their needs expensive, but, since these services often rely on prescriptions rather than addressing the clinical cause, can contribute to antimicrobial resistance.

**Methods:**

The RETURN intervention has been developed with substantial community co-production, to be delivered opportunistically in urgent dental care settings. This brief intervention is delivered by dental nurses and involves material relevant to the ‘in-group’ targeted. The material includes booklets relating to barriers to planned dental visiting with corresponding short video clips featuring local people and including a modelling element. Dental nurses are trained to have supportive and non-judgemental conversations, assisting patients to set personal goals and action plans, which are reinforced in a follow-up text within a few weeks. A randomised controlled trial will be undertaken in 3 types of sites: dental practices delivering urgent care (a) within working hours, (b) out of hours, and (c) in a Dental Hospital. The trial will recruit 1180 adult urgent dental care users over 12 months, who have not visited a dentist for a planned care appointment for 2 years or more and do not have a dentist who they visit for routine care. It aims to investigate the effectiveness and cost-effectiveness of the intervention and to explore whether the intervention has different effects across the socio-economic gradient. Participants will be followed up at 6, 12 and 18 months after randomisation. Co-primary outcomes are attendance at a dental practice for planned care within 12 months and self-reported oral health-related quality of life at 12 months.

**Discussion:**

This is a pragmatic trial, evaluating the effectiveness of the intervention under the usual condition in which it might be applied. Since dental practices work as independent contractors to the NHS, this brings implementation and fidelity challenges which will be explored and described in embedded qualitative work.

**Trial registration:**

ISRCTN registry identifier ISRCTN84666712. Registered 12/04/2021.

## Administrative information

Note: the numbers in curly brackets in this protocol refer to SPIRIT checklist item numbers. The order of the items has been modified to group similar items (see http://www.equator-network.org/reporting-guidelines/spirit-2013-statement-defining-standard-protocol-items-for-clinical-trials/).

**Table:** 

Title {1}	Behavioural intervention to promote the uptake of planned care in urgent dental care attenders: study protocol for the RETURN randomised controlled trial
Trial registration {2a and 2b}.	ISRCTN registry. Trial identifier ISRCTN84666712. Registered 12/04/2021
Protocol version {3}	Version 4.0 08/12/2021.
Funding {4}	Funded by the NIHR Programme Grant for Applied Research (project number RP-PG-0616-20004)
Author details {5a}	Affiliations of authors: University of Liverpool, Liverpool UK (Harris, Lowers, Burnside, Best, Van der Zande, Maitland); University of Exeter, Exeter, UK (Hulme); University of Dundee, Dundee, UK (Clarkson); Staffordshire University, Stoke-on-Trent, UK (Cooke)
Name and contact information for the trial sponsor {5b}	Research Support Office University of Liverpool 2nd Floor Block D Waterhouse Building 3 Brownlow Street Liverpool L69 3GL Tel: 0151 794 8739 Email: sponsor@liverpool.ac.uk
Role of sponsor {5c}	The University of Liverpool sponsor has overall responsibility to ensure proportionate, effective arrangements are in place to set up, run and report the research project such as ensuring that clear agreements are reached, documented and carried out, respecting the dignity, rights, safety and wellbeing of participants and the relationship with healthcare professionals. A Programme Steering Committee (PSC) appointed by the NIHR funder reviews the conduct and progress of the study against the research plan, timelines and objectives agreed with the funder, and so may advise on study design and conduct. The funder assures the quality of the trial, taking the lead in establishing that the research proposal is worthwhile, of high scientific quality, has an appropriate research infrastructure with expert clinical trial management, has the capacity to comply with the principles of GCP and represents good value for money. The sponsor, PSC and NIHR funder do not have a role in data collection, analysis, writing of the report; or the decision to submit the report for publication

## Introduction

### Background and rationale {6a}

Dental diseases are both very common and expensive to treat, with costs amounting to an estimated $356.80 billion in treatment expenditures and $187.61 billion in productivity losses worldwide [1]. Unfortunately, an inverse care law exists whereby those who have the highest dental needs are the least likely to receive good quality care—a pattern which is both considerable and globally consistent [2, 3]. A social patterning of dental attendance exists where those from more deprived socio-economic backgrounds are less likely to visit a dentist for planned visits [3–5], even though poorer oral health disproportionately affects those at the lower end of the socio-economic spectrum [6–8]. Several studies have shown that socio-economic inequalities in oral health are, at least some extent, mediated by dental attendance [9–11]. A large body of evidence, including longitudinal studies, show that dental attendance is linked to better oral health [4, 12, 13]. Patients who are regular dental attenders are also more likely to report fewer negative impacts of poor oral health on their quality of life [5].

As many as a third of the UK population report that they only attend a dentist when they have a problem, or never do [14]. While early studies tend to adopt a dichotomous distinction between regular and irregular attenders [6], often based on dental attendance over the preceding 12 months, more recent studies have started to look at patterns in dental attendance in terms of the lifecourse [5, 15–17]. These studies show that only a minority of patients maintain dental attendance patterns consistently throughout their lives, and a small minority never visit a dentist. Consistent planned dental visits over the long term have been shown to be associated with better oral health, compared to patients who do not visit a dentist at present, but used to do so, or patients who have never regularly attended dental care services [5, 15, 17].

Planned care refers to services for pre-arranged appointments as opposed to urgent care appointments. Planned care appointments are arranged relating to either an examination or check-up or a course of treatment (where further care is identified as needed following an examination). Regular check-ups are recommended so that an early diagnosis of oral disease can be made (dental caries, periodontal disease, oral cancer). These check-ups also allow for the detection of the oral manifestations of systemic disease and appropriate referral and establishing a therapeutic alliance (relationship) between the provider and patient which helps support patients’ adherence to preventive recommendations [18]. As well as monitoring patients’ oral health and allowing secondary prevention (limiting progression and effect of oral diseases at an early a stage, by, for example applying fluoride varnish to early demineralised lesions), check-up visits often incorporate a third role in primary prevention—influencing patient behaviour with a view to preventing oral diseases before they occur, where members of the dental team give advice to help patients improve their tooth-brushing and dietary behaviours and stop smoking [18].

What constitutes ‘regular’ visits in terms of the optimal frequency for visiting a dentist for a check-up has been the topic of considerable debate, with recommendations varying from 6-monthly examinations (recall visits) to longer periods tailored to the level of patients’ risk of developing problems (usually up to 2 years for people with low risk of disease) [18, 19]. Since there is general agreement that planned dental visits at least every 2 years are beneficial for monitoring purposes with prevention and treatment as needed; we define planned dental visiting behaviour as having visited a dentist in the past 24 months for a non-urgent appointment.

People with irregular attendance patterns often tend to be those who wait until there is a problem such as toothache and swelling which impacts their quality of life [20]. More than 25% of UK adults report they had dental pain over the last 12 months, and this is disproportionally affects people with manual backgrounds [21]. Patients attending for a dental emergency have often been in pain for over 2 weeks, and a significant proportion of those attending dental emergency services, do so repeatedly for the same condition [22]. Some people, even when in pain, avoid using dental services and visit other services such as General Practice (GP) or Accident and Emergency (A&E) departments instead, which increases the costs of meeting their dental needs and puts an added burden on these often stretched services [23].

Since General Practitioners (GPs) prescribe antibiotics for over half of patients who attend their services with dental problems, this, together with the high level of antibiotics prescribed in dental practice for people attending with urgent problems, contributes to the problem of antimicrobial resistance [24–27]. In addition, there are potentially serious consequences if dental infections remain untreated, such as sepsis, which may require hospitalisation, resulting in high costs to the National Health Service (NHS) [28]. In short, a lack of planned dental visiting can result in dental visits for acute dental problems which is expensive and of limited effectiveness—geared towards giving only short-term relief of symptoms [29].

A brief psychological intervention (the RETURN intervention) has been developed to be delivered to adults attending dental services for urgent care which aims to promote planned dental visiting. The intervention has been developed using extensive qualitative work [16] and with patient and public involvement to identify salient barriers to planned dental care. This work also helped to develop appropriate imagery and narrative for the intervention material, including video stories from local people which accord with the ‘in group’ identity of the population targeted. This is in recognition of identity-based motivation theory which explains that people are most likely to be motivated and act towards goals if they see that the action is in congruence with their perceived self-identity [30].

In order to promote planned dental visiting, and through this to improve oral health, and reduce impacts of oral disease such as toothache and use of antibiotics for dental problems [29], the intervention uses multiple behaviour change techniques. The intervention is delivered by dental nurses who have been trained to discuss the intervention material with patients using active listening techniques such as relevant open questions, non-judgemental language, non-directive talk and acknowledging patient’s priorities, beliefs and challenges. The intervention is aimed at helping patients overcome their barriers to dental attendance for planned care and includes a ‘goal setting and action plan’ element [31]. Goal setting (a distal goal, e.g. attending for planned dental care) and creating an action plan to achieve it may help patients overcome significant barriers preventing behaviour change. Action planning involves proximal goals; in this case, these smaller achievable short-term goals would be designed to overcome a patient’s specific barrier(s). These techniques are simple to administer and are known to assist people with achieving health-related behaviour change [32]. The delivery of the RETURN intervention has been previously tested in a feasibility study. There are no known risks of the intervention, to either the trial participants, or the trial research teams, although there is a possibility that participants experience a heightened dental anxiety after thinking in detail about visiting a dentist. Possible benefits of the intervention are improved oral health and quality of life after taking up planned dental care, with avoidance of dental emergencies and its impact on the cost to the NHS.

### Objectives {7}

The overall objective of this trial is to assess the effectiveness and cost-effectiveness of the RETURN intervention for urgent dental care service users and to explore whether the RETURN intervention has different effects across the socio-economic gradient.

#### Primary objectives

1. To identify whether there is a difference between intervention and control groups in rates of attendance for a planned care appointment at a dental practice within 12 months (as measured by NHS Business Services Authority [BSA] data).
2. To identify whether there is a difference in oral health-related quality of life 12 months after randomisation.

#### Secondary objectives

1. To identify whether the behavioural intervention has different effects across the socio-economic gradient.
2. To identify the psychological factors which mediate any intervention effect.
3. To identify whether there is a difference between intervention and control groups in self-reported rates of attendance and/or attempts to make an appointment at a dental practice for planned dental care during the follow-up period.
4. To identify whether there is a difference between intervention and control groups in rates of attendance at a dental practice (as measured by BSA data) for a planned care appointment within 18 months.
5. To identify whether there is a difference between intervention and control groups in oral health quality of life at 6 and 18 months.
6. To identify whether there is a difference between intervention and control groups in halitosis and bad taste as indicators of dental infection at 6, 12 and 18 months.
7. To identify whether there is a difference between intervention and control groups in clinical dental treatment received at 6, 12 and 18 months.
8. To identify whether there is a difference between intervention and control groups in self-reported rates of attendance for urgent dental care at A&E, GP practices and urgent dental care services at 6, 12 and 18 months.
9. To identify whether there is a difference between intervention and control groups in rates of antibiotics or analgesics for dental problems prescribed by a dentist, GP Accident and Emergency department or walk-in centre at 6, 12 and 18 months.
10. To monitor any change in dental anxiety after receiving the intervention.
11. To estimate the cost effectiveness of the intervention compared with usual care at 12 and 18 months.

### Trial design {8}

The RETURN trial is a randomised, open-label, superiority study involving delivery of an opportunistic psychological intervention to urgent dental care users developed to increase the uptake of planned dental care and reduce health inequalities. The allocation ratio between intervention and control is 1:1. The trial will recruit 1180 urgent dental care users over 12 months. Participants will be followed up at 6, 12 and 18 months after randomisation.

## Methods: participants, intervention and outcomes

### Study setting {9}

Recruitment will be in three site types: a teaching Dental Hospital (Liverpool University Hospitals NHS Foundation Trust); NHS dental practices providing care out-of-hours; and NHS dental practices providing in-hours urgent care, all located in Cheshire and Merseyside which is in the North West of England, UK. A list of sites can be obtained from the research team.

### Eligibility criteria {10}

#### Inclusion criteria

Patients eligible for the trial must comply with all of the following at randomisation:

1. Adults (aged 18 years or over) seeking urgent dental care.
2. Has not visited an NHS or Private dentist for a non-emergency appointment (i.e. when not in pain or symptomatic) for 2 years or more, and do not have a dentist who they visit regularly for routine care.
3. Able to provide either a telephone number, email or postal address to allow follow-up.
4. Has provided written consent.
5. Has adequate understanding of spoken and written English.
6. Are responsible for making their own dental appointments, i.e. not done by a carer.

#### Exclusion criteria

Any patient meeting any of the criteria listed below at baseline will be excluded from trial participation:

1. Have previously been enrolled in the RETURN feasibility or main trial.
2. Lives with, or related to, a participant in the RETURN feasibility or main trial.

Patients who go on to have planned care with undergraduate students in the Dental Hospital will not be excluded, although the route that patients’ take to planned care will be reported.

### Who will take informed consent? {26a}

Following screening, consent will be sought from potentially eligible patients by RETURN trained staff, who are delegated and qualified to do so. This includes dental nurses in urgent care services who have received training in taking this consent and are observed to reach competency in this by a member of the research team.

### Additional consent provisions for collection and use of participant data and biological specimens {26b}

Participants will be given the option in the consent form, to opt into or out of consenting to their data potentially being used in future studies. No biological specimens are being collected in this study.

### Intervention

#### Explanation for the choice of comparators {6b}

Participants will receive the usual care that is provided by the site they were recruited at.

#### Intervention description {11a}

Participants randomised to the RETURN intervention will receive a booklet pack providing educational resources to help overcome six common dental visiting barriers (‘I don’t have time’, ‘I don’t think to go when I’m not in pain,’ ‘I don’t have trust in dentists’, Cost, Embarrassment, Anxiety). The booklet pack contains a set of resources to help participants prepare for and support their attendance for a planned dental appointment, such as information on the cost of treatment and how to claim for free care if in an exempt from charges group; relaxation exercises which they might use when waiting for their dental appointment, and a credit card sized information card, which the participant can give to their employer advising that further dental care appointments are recommended. The pack contains some persuasive communication material, such as information about the consequences and costs of delaying care until they have toothache. A leaflet describing how dental services operate during the COVID pandemic will also be included, including for example having to wait outside the practice until just before their dental appointment time.

A trained dental nurse or research dental nurse (trained dental nurses employed by the research team) will help the participant identify a barrier which is most important to them using behaviour change conversational techniques such as empathic listening, non-judgement and non-directive talk. The participant will then view an online video clip involving a story from someone who has experienced this barrier, which is overlaid with an animation to make it especially engaging, and relevant to the ‘in-group’ targeted [30]. The intervention contains elements of ‘modelling’. Both video stories and quotes in the written material are generated from qualitative and public involvement work which underpinned the development of the intervention, with many of the video stories featuring members of a community advisory group.

While in the dental setting, using the booklet material, participants will make a goal regarding attending for a planned dental appointment, and an action plan that will help them overcome their chosen barrier to dental visiting. These will be photographed by the dental nurse on the Tablet PC and uploaded to the trial database to facilitate a follow-up text which will be sent within 2 weeks of the participant receiving the intervention. For participants who discuss more than one barrier within a delivery session, encouragement will be provided by the trained dental nurse to look at relevant intervention materials at home. Participants will receive a follow-up text with an online link to the material and videos which will be accessed via a restricted access web portal, and with a supportive message around the participant’s goal and action plan(s) recorded.

#### Criteria for discontinuing or modifying allocated interventions {11b}

Once patients are randomised to receive the intervention, there will be no modification of intervention content.

#### Strategies to improve adherence to interventions {11c}

A key component of the intervention is the conversation between the person delivering the intervention and the patient, which is to be conducted in a non-judgemental and supportive manner. All staff delivering the intervention will be trained according to a written intervention delivery manual. Training of staff will include both didactic teaching and role play, followed by a period of shadowing of intervention delivery (the member of staff first observes the intervention being delivered in situ, and then delivers it themselves while being observed by a member of the research team). An observation checklist containing essential elements of the intervention to be included is completed. This covers five essential domains: (i) empathetic listening with non-directive and non-judgement talk; (ii) elicitation and discussion of barriers to planned dental visiting leading to introduction of the booklet pack; (iii) being shown a video clip with patients’ reflection on how this relates to their own circumstance; (iv) highlighting relevant aspects of the booklet pack relevant to the patient’s circumstances, emphasising benefits of planned dental visits, hope for the future and assurance about the patients’ capability of overcoming relevant barriers; and (v) setting of a planned dental visiting goal and action plan relating to at least one barrier which is specific, measurable, achievable, realistic and anchored within a time frame (SMART). When the member of staff is observed to deliver at least 80% of these elements contained under each domain of the intervention in situ, they are deemed to be competent to deliver the intervention independently. In order to ensure consistency between raters of intervention delivery competency, a calibration exercise will take place using audio recordings of delivery sessions undertaken by RETURN research nurses, independent observation checklist scoring, comparison and group discussion. The exercise will be repeated until all discrepancies in scoring are resolved. A sign off guidance document will be produced to facilitate consistent sign off decisions.

In order to monitor fidelity of intervention delivery, all intervention delivery sessions will be audiotaped, with consent from both the patient and dental nurse. Recordings will be collected from research sites on a monthly basis throughout the recruitment period. Each month, 20% of the recordings from each dental nurse will be randomly selected and independently rated by two members of the research team using the observation checklist outlined above. Eighty percent of the necessary components will need to be present in each recording for the intervention delivery session to be deemed as reaching an acceptable fidelity threshold. If there is disagreement between the two raters, this will be resolved through discussion until agreement is reached. Where necessary, a third rater will be introduced to resolve disputes in scoring. In each instance where a recording is found to not have reached the fidelity threshold of 80%, feedback will be provided to the dental nurse in question using supervision sessions. Thereafter, a necessary refresher training and further shadowing will be undertaken if any member of staff delivering the intervention is found not to be delivering the key intervention ingredients consistently.

#### Relevant concomitant care permitted or prohibited during the trial {11d}

To avoid potentially confounding issues, co-enrolment in other dental trials is discouraged, although will be considered if viewed by the research team as not having a potential impact on the dental care or oral health of RETURN participants.

#### Provisions for post-trial care {30}

There are no anticipated harms from trial participation and therefore no arrangements for post-trial care.

### Outcomes {12}

#### Primary outcomes

1. Attendance at a dental practice for a planned care appointment within 12 months, collected from NHS Business Services Authority (BSA) data.
2. Self-reported oral health-related quality of life as measured by the value at 12 months of the summary score of the short form Oral Health Impact Profile (OHIP-14) [33].

#### Secondary outcomes

1. Attendance at a dental practice for a planned care appointment, self-reported from phone calls at 6, 12 and 18 months.
2. Attempt to make a planned care appointment, self-reported from phone calls at 6, 12 and 18 months.
3. Attendance at a dental practice for a planned care appointment within 18 months, collected from BSA data.
4. Self-reported oral health-related quality of life, measured by the final value at 6 and 18 months adjusting for the baseline value, of the summary score of OHIP-14 [33].
5. Urgent attendance for dental care at dental practice, A&E or Dental Hospital. Patient self-reported at 6, 12 and 18 months.
6. Halitosis and bad taste, measured by patient self-report at 6, 12 and 18 months.
7. Treatment received, as measured by BSA clinical dataset, Dental Hospital audit and patient self-report at 6, 12 and 18 months.
8. Antibiotic prescription by dentists, measured by BSA data, Dental Hospital audit and patient self-report at 6, 12 and 18 months.
9. Antibiotic prescription by GP/A&E/Walk-in centre measured by patient self-report at 6, 12 and 18 months.
10. Analgesic prescription, measured by patient self-report and Dental Hospital data at 6, 12 and 18 months.
11. (safety): Dental anxiety, as measured by the change in the continuous score of the Modified Dental Anxiety Scale (MDAS) [32] recorded in patient self-report at 6, 12 and 18 months.
12. Incremental cost-effectiveness ratio at 12 and 18 months derived from BSA data, patient self-report resource use and EQ-5D-5L at baseline, 6, 12 and 18 months.

Where an outcome measure (secondary outcomes 7, 8 and 10) using data from different sources produces conflicting results, e.g. NHS BSA and patient self-reports on treatment received, the outcome will be recorded as being present if recorded by either data source. Patient self-report of dental services used will include private as well as NHS services, whereas BSA data only records NHS services used.

### Participant timeline {13}

**Fig. 1:**
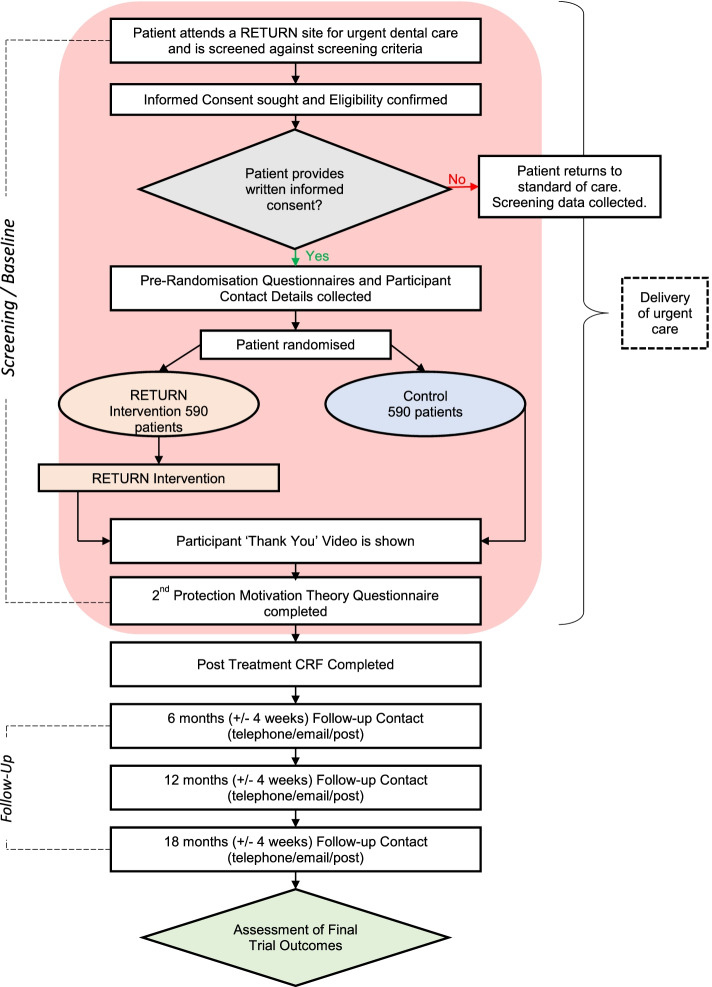
Flow diagram of trial design

Figure 1 provides a flow diagram of trial design. This is a pragmatic trial of an opportunistic intervention, and so all trial processes may be undertaken either before or after the receipt of the clinical consultation and treatment (urgent dental care).

#### Participant identification and screening

A banner/poster in the dental service will inform patients that a research study is taking place at the site. Patients potentially eligible to participate will be given an A5 leaflet by a dental nurse when they arrive at their appointment to explain that this study is taking place. A dental nurse will routinely ask questions about the patient’s age and ability to understand English and to determine whether a patient has their own dentist. During this initial discussion, if a patient does not fulfil the eligibility criteria, then the reasons will be recorded on a screening log.

#### Informed consent

Written informed consent will then be sought from potentially eligible patients following discussion of objectives, risks and inconveniences of the trial. A NHS Research Ethics Committee (REC) approved Participant Information Sheet and Consent form (PISC) will be used to prompt discussion with the patient and answering any questions. The PISC will emphasise that participation in the trial is voluntary and that the patient may withdraw from the trial at any time and for any reason.

#### Eligibility assessment and confirmation

A trained member of staff will complete an eligibility assessment to confirm eligibility, prior to completing the baseline assessments and randomising the patient. Confirmation of eligibility will be recorded on a case report form (CRF) and on a participant log.

#### Baseline assessments

The following data will be collected by participant self-report prior to randomisation:

- Demographic information:
  - Sex (Male/female/prefer not to say)
  - Age
  - Ethnicity
- Socio-economic information:
  - Education level
  - Employment status:
    - In paid employment: Yes/No
    - If not employed whether: currently looking/ student /retired /not able due to disability/ not currently looking/ other
  - Benefit status: Yes/No/Prefer not to say
- OHIP-14: 14-item oral health-related quality of life questionnaire [33]
- EQ-5D-5L: 5-item generic quality of life questionnaire [34]
- Modified Dental Anxiety Scale (MDAS): 5-part dental anxiety questionnaire [35]
- Protection Motivation Theory: questionnaire measuring psychological mediators [36]
  - Susceptibility of developing urgent dental problems
  - Response efficacy for attending for planned care – that this would reduce the chance of developing an urgent dental problem
  - Severity – not attending for planned dental care means they would be at risk of tooth loss, bad breath, or getting an abscess
  - Self-efficacy of attending for planned dental care
  - Intention to attend for a planned care appointment
- Self-report of halitosis [37]
- Self-report of bad taste [38]
- Prescription information—prescription of antibiotics and/or analgesics for dental problems by a dental, GP or A&E or similar centre within the last 6 months.

#### Protection motivation theory: psychological mediation

The Protection Motivation Theory (PMT) is based on a premise that there are two alternative responses to threats (such as dental pain) which are either protective or adaptive coping strategies, or maladaptive responses such as avoidance or procrastination [39]. PMT predicts that the primary determinant of adaptive coping strategies such as planned dental visiting is whether or not an individual has formed a protection motivation with regard to the focal behaviour: for example, an intention to act to manage a health threat (e.g. dental pain). This is in turn informed by parallel appraisal processes: threat appraisal and coping appraisal. Threat appraisal variable predictors comprise perceived severity (seriousness) of the health threat, perceived susceptibility (vulnerability) and fear of the health threat. Applied to dental attendance, patients will be asked to consider the severity of their dental pain and their susceptibility for experiencing dental pain. In order to minimise participant burden; MDAS scores will be used as an indicator of fear of the health threat [35]. Coping appraisal variables comprise self-efficacy (i.e. confidence in performing the focal behaviour) and response efficacy. Applied to dental attendance, patients report their confidence they can attend dental appointments and that such attendance would be an effective way to reduce the threat of dental pain

#### Halitosis and bad taste

Since collection of clinical outcomes is beyond the scope of the trial, self-reported halitosis and bad taste provide an indicator of a discharging dental abscesses, poor oral hygiene and periodontal disease [37, 38]. A binary variable will be created for each time point to represent if there are indicators of dental infection based on the answers to the halitosis and bad breath questions (1=Any indicator of dental infection, 0=No indicators or dental infection). To indicate dental infection based on the halitosis questions, the participant must answer the questions in the following way:

- In the last 6 months, have you noticed a bad taste in your mouth? Answer: Yes

Then the participant must answer ‘Yes’ to either of the following questions:

- If you told us you have noticed a bad taste, how much does it bother you? Answer: Either very bothered or extremely bothered
- If you told us that you have ever noticed a bad taste in your mouth, does it affect your social life or your work life? Answer: Yes

To indicate dental infection based on the bad breath questions, the participant must answer these questions in the following way:

- Within the last 6 months, have you noticed you have bad breath? Answer: Yes

Then the participant must also answer ‘Yes’ to either one of the next questions

- If you told us that you have ever noticed bad breath, how much does it bother you? Answer: very bothered or extremely bothered
- If you told us that you have ever noticed bad breath, does it affect your social life or your work life? Answer: Yes

However, the participants must also answer the final question in the following way to determine that indicators of dental infection are present and are not from other causes:

- Have you had tests or treatment from medical staff for this (bad breath) such as an examination of your throat, sinuses, stomach or blood tests? Answer: No

To determine indicators of dental infection overall, both the halitosis and bad breath indicators should be present.

### Sample size {14}

Twenty six percent of patients attending urgent dental care access centres report they feel attending a dentist regularly is important [40] and other work shows that only 20% of users of such services have their own dentist [41]. Since unpublished data from an NHS evaluation in the North West of England found that an average of 16% of users of urgent dental care services delivered in dental practices returned to a practice for planned dental care over the subsequent 22 weeks, we estimated that over the longer period of 12 months, we might expect 30% of urgent dental care users to return for a planned dental care appointment. The calculation is based on a minimum clinically important difference of 10 percentage points (i.e. an improvement to 40% of patients attending a dentist) in the first of the joint primary outcomes. We estimate there is a possibility of some contamination between patients within practices. Rather than use a cluster randomised design, we have reduced the difference we want to detect to 9 percentage points to allow for potential contamination. Based on this, a sample size of 559 per group would give 80% power at *α* = 0.025 (to allow for joint primary outcomes) to detect this improvement in the intervention group. Allowing for 5% loss to follow-up gives a total target sample size of 590 per group.

For the second primary outcome, OHIP-14, we estimate a standard deviation of 11 points at 1-year follow-up. We also assume a higher rate of loss to follow-up of 20% for this outcome, as it will require successful telephone follow-up. Based on this, our total sample size of 1180 will give 80% power to detect a minimum clinically important difference of 2.25 points, with *α* = 0.025.

### Recruitment {15}

Participants in both intervention and control groups will be given a total of £50 in shopping vouchers in reimbursement for their time participating in the trial. The baseline voucher of £15 will be given to participants at their baseline visit, subsequent vouchers will be provided to the participants’ home address. Although there will be no competitive recruitment targets for sites, the research team will produce a newsletter to update sites on recruitment rates in different sites once site initiation and recruitment has commenced. The research team will also maintain regular contact with trial sites throughout the recruitment period.

### Assignment of interventions: allocation

#### Sequence generation {16a}

Participants will be equally randomised to the intervention or control group in a 1:1 ratio using a secure (24-hr) web-based randomisation programme controlled centrally by LCTC. Randomisation lists will be generated using block randomisation with random variable block length, stratified by site. The lists will be produced by an independent statistician (who is not otherwise involved in the RETURN trial) at the Liverpool Clinical Trials Centre (LCTC).

#### Concealment mechanism {16b}

Participant allocations will be irrevocably released upon completion of the web-based randomisation form by a dental nurse. Allocation concealment will be ensured as the service will not release the randomisation code until the patient has been recruited into the trial. This takes place after all baseline measurements have been completed.

#### Implementation {16c}

A trained member of staff (dental nurse) will use the web-based randomisation system to randomise the participant which will release the next pre-generated allocation from the allocation sequence to enroll participants, and assign participants to the intervention or control arm of the study.

### Assignment of interventions: blinding

#### Who will be blinded {17a}

RETURN is an open trial and as such investigators involved in the delivery of the intervention and participants in the trial are not blind to allocations. The statistical analysis plan will be written prior to the statistical team having access to any unblinded trial data.

#### Procedure for unblinding if needed {17b}

This is not applicable since patients and trial staff are unblinded to the allocations already.

### Data collection and management

#### Plans for assessment and collection of outcomes {18a}

Table 1 provides a summary of procedures and assessments relative to participant timelines. Patients receiving dental care in an NHS dental practice following baseline will have a centrally held record in data held by NHS BSA, which will require participant IDs collected at baseline to be matched with participant NHS BSA identifiers. To maximise matching of participant descriptors with routinely held NHS BSA follow-up data, participant postcodes will be checked at each follow-up point in case this has changed since baseline. For participants receiving dental care in the Dental Hospital (DH) following their baseline data, details will be retrieved from clinical records held in the Dental Hospital identified by their unique RQ number. This follow-up data will be collected using a CRF proforma and conducted as an audit of participant DH records. Follow-up data collected by telephone calls by the central research team will be audiotaped and 10% checked by a different researcher than the researcher entering the data during the telephone call. Where disagreements are identified, these will be addressed and resolved by agreement, and any data entry errors rectified. Data collection forms can be obtained from the research team.

**Table 1:** Schedule of processes and assessments

Procedures		Screening /Baseline	RETURN Intervention Group	Control Group	Follow-up		
					6 months (+/− 4 weeks)	12 months (+/− 4 weeks)	18 months (+/− 4 weeks)
Assessment of Eligibility Criteria		X					
Signed consent form		X					
Urgent dental treatment (trial processes and intervention around delivery of urgent care)		(X)	(X)	(X)			
Collection of Contact Details, including Home Postcode (to determine IMD as a mediator)		X					
Verification of NHS BSA Matching Descriptors					X^d^	X^d^	X^d^
Collection of RQ6 number^a^		X					
***Self-Reported Assessments***	Socio-economic status (Education level, employment and benefit status)	X			X^b^	X^b^	X^b^
	Age	X					
	Gender (including ‘prefer not to say’)	X					
	Ethnicity	X					
	OHIP-14	X			X	X	X
	Halitosis and Bad Taste	X			X	X	X
	EQ-5D-5L questionnaire	X			X	X	X
	Protection motivation theory questionnaire	X	X^c^	X^c^	X	X	
	Modified dental anxiety scale (MDAS)	X			X	X	X
	Use of Routine Dental Practices and Type (NHS or Private)	X			X	X	X
	Use of Student Teaching Pathway (Dental Hospitals)	X			X	X	X
	Use of Dental Care Practices, including access centres)	X			X	X	X
	Attendance at a dental service at baseline, and any subsequent urgent, or planned, dental care at follow-up	X			X	X	X
	Use of General Medical Practice for Urgent Dental Care	X			X	X	X
	Use of A&E for Urgent Dental Care	X			X	X	X
	Hospital In-patient Care for Dental Problems	X			X	X	X
	Hospital Out-patient Care, including Dental Hospitals	X			X	X	X
	Prescription of antibiotics and/or analgesics within the last 6 months	X			X	X	X
	Health Economics Participant Questionnaire (including cost of dental treatment, expenses due to dental care, days off work, travel expenses, non-prescription medications [CSRI])				X	X	X
Participant randomised and allocation provided		X					
Administration of RETURN Intervention			X				
Record Intervention Delivery Timing (before, or after, urgent care treatment)			X				
Participant’s Goal and Action Plan			X				
Participant ‘Thank you’ video			X	X			
Participant Voucher Reimbursement			X	X	X	X	X
***Dental team***	Date of urgent dental care visit, participant sex and date of birth.	X					

^a^Participants recruited in Dental Hospital only

^b^Employment status only

^c^RETURN Intervention and Control Groups will complete second protection motivation theory questionnaire after being shown the Thank you video

^d^Participant name, date of birth and postcode. If participant postcode has changed from baseline, then the month that the participant changed their address will also be collected

#### Plans to promote participant retention and complete follow-up {18b}

Participants in the intervention and control arm will be shown a short, animated video thanking them for participating in the trial while in the dental setting. The ‘Thank you’ video gives information on what to expect in subsequent follow-up contacts in 6, 12 and 18 months’ time and aims to maximise retention in both control and intervention arms. Shopping vouchers for reimbursement for participants’ time participating in the trial will be posted to their home address and connected to key follow-up contact points (£10 at the 6- and 18-month follow-ups and £15 at the 12-month follow-up point.

#### Data management {19}

Site-specific trial-related information will be stored securely and confidentially at sites and all local relevant data protection policies will be adhered to. Follow-up of participants by phone, email or letter require that participant contact details including name, email address and telephone details are stored. Contact information will be added to the RETURN contact database at trial enrolment and the contact database will be maintained securely on password-protected computers. A data sharing agreement will be in place between the University of Liverpool and NHS BSA to safeguard the confidentiality of personal identifiable data transfer between the two organisations. Leeds University is also considered to be a Joint Data Controller, with respect to the required data for Health Economics analysis. Data will be transferred using a Secure File Transfer platform.

Data queries will be sent via email and through REDCap (the data collection database used in the trial) to site staff. The person responsible for dealing with the data query will be the site Principal Investigator (PI).

### Confidentiality {27}

This trial will collect personal data (e.g. participant names), including special category personal data (i.e. data concerning health) which will be handled in accordance with all applicable data protection legislation. Individual participant health information obtained will be considered confidential and not disclosed to third parties. Case Report Forms will be labelled with the patient’s initials and a unique trial screening and/or randomisation number. Consent forms will be transferred separately to any other trial documentation to ensure the pseudonymisation of special category data is maintained.

#### Plans for collection, laboratory evaluation and storage of biological specimens for genetic or molecular analysis in this trial / future use {33}

No biological specimens are collected in this trial.

### Statistical methods

#### Statistical methods for primary and secondary outcomes {20a}

An extract of the NHS BSA data will be obtained after 12 months (52 weeks) of recruitment. This will aid in the preparation of analysis code before receiving the 18-month (78 weeks) data. Final analysis of the first primary outcome will be conducted on the 18-month data. Analysis of the first primary outcome will involve the number and percentage of participants found to have a planned dental appointment within 12 months which will be reported for each treatment group separately. The denominator will be the number of randomised participants in each treatment group. A comparison between groups will be done using logistic regression, adjusted for the stratification factor site as a random effect (random intercept only). The derived binary variable for attendance for a planned appointment within 12 months will be the dependent variable. The odds ratio and corresponding 97.5% confidence interval will be reported and *p*-value. A sensitivity analysis will be conducted removing all participants from the above primary analysis who were recruited in dental practices (i.e. not randomised at the Dental Hospital) and who do not have a matched NHS BSA record at baseline. This is to assess the impact of including the participants who were not successfully matched in the NHS BSA data at baseline which may indicate that it was possible to get NHS BSA data at later timepoints.

Analysis of the second primary outcome will involve summaries of OHIP-14 scores presented at baseline and 12 months separated by treatment group, for all participants included in this analysis. The OHIP-14 scores at 12 months will be analysed using linear regression, adjusting for the baseline value of OHIP-14 and the stratification factor site as a random effect (random intercept only). The estimated treatment effect and corresponding 97.5% CI will be reported along with the *p*-value. This will be a complete case analysis, only participants with valid baseline and 12-month questionnaires will be included in the analysis.

A second analysis will also adjust for other pre-specified baseline variables, including dental anxiety and socio-economic status.

Analyses will include all randomised participants. The principle of intention-to-treat, as far as practically possible, will be the main strategy of the analysis adopted for primary outcomes and all the secondary outcomes. To allow for joint primary outcomes, the type I error rate will be controlled by setting *α* = 0.025 and therefore confidence intervals (CI) will be presented at 97.5% for both the primary outcomes. No other adjustments will be made for multiplicity. Other secondary outcomes will be reported at the 95% confidence level. *p*-values will be reported to 3 decimal places or 1 significant figure if *p*<0.001. Percentages will be reported to 1 decimal place throughout; continuous baseline characteristics will be presented to 1 decimal place or 1 significant figure and all other values will be present to 2 decimal places or 1 significant figure. All analyses will be performed with standard statistical software (SAS version 9.4 or later). For the purpose of measuring timepoints, 6 months = 26 weeks, 12 months = 52 weeks and 18 months = 78 weeks. A full statistical analysis plan is available from the research team.

#### Interim analyses {21b}

No formal interim analysis is planned.

#### Methods for additional analyses (e.g. subgroup analyses) {20b}

Whether there is a differential intervention effect over the socio-economic gradient will be explored. The dependent variable of this analysis will be defined in the same way as was defined for primary and secondary outcomes. This outcome will be analysed in the same way as other outcomes; however, the estimated treatment effect and corresponding 95% CI will be reported (instead of a 97.5% CI) along with the *p*-value. However, the logistic regression model will include IMD, and an interaction between IMD and intervention and an interaction between IMD and site. Other socio-economic factors such as employment status may also be considered, as well as gender and ethnicity, while acknowledging the potential for multicollinearity.

A further analysis of the primary outcome (using logistic regression) will also adjust for the following baseline covariates:

- Education level (‘No formal qualifications’ will be the reference category)
- Index of multiple deprivation (IMD)
- Modified dental anxiety scale (MDAS)

The baseline covariates of education and IMD will be assessed for multicollinearity as they may be correlated. If it is found that there is an association between them, then one of them will be removed.

#### Methods in analysis to handle protocol non-adherence and any statistical methods to handle missing data {20c}

Protocol deviations will be defined in the RETURN main trial monitoring plan and specified as minor or major. For final analyses, the number of participants experiencing each protocol deviation will be presented split by allocation group, along with the numbers with at least one minor deviation, at least one major deviation and at least one deviation of either classification. No formal statistical testing will be conducted. The main approach to analysis of outcomes will follow the intention-to-treat principle.

#### Plans to give access to the full protocol, participant-level data and statistical code {31c}

The main trial protocol will be available. Sharing of fully anonymized patient-level data and statistical code would be considered on request.

### Analysis of psychological variables

Analysis involving psychological variables will be undertaken at 12 and 18 months to investigate differences between intervention and usual care in perceived susceptibility, severity, response efficacy, self-efficacy, fear (MDAS) and intentions. Independent groups *t*-tests will be conducted to test differences between groups in these variables. To control for the increased type 1 error rate due to multiple comparisons, the significance level (*p* < 0.05) will be divided by the number of comparisons (6); this means adopting a significance level of *p* < 0.008 to indicate significant differences between groups. If significant differences in psychological variables are found between groups, mediation analyses will be undertaken. One of two types of mediation analysis will be performed depending on *t*-test results. If differences are only found in intentions, then a simple mediation analysis of intervention>intention>planned dental attendance will be undertaken. If differences are found in any of the other PMT variables then, consistent with PMT proposals, serial mediation will be undertaken: Intervention > PMT variable(s) > Intentions > planned dental attendance. All mediation analyses will be conducted using version 4 of PROCESS macro in SPSS 28 [42].

### Health economics analysis

The cost-effectiveness analysis will be undertaken at 12 and 18 months. The primary outcome of the cost-effectiveness analysis will be cost per quality-adjusted life years (QALYs). Utility values will be obtained from participants’ responses to the EQ-5D-5L questionnaire completed at baseline and 6, 12 and 18 months. The derived utilities will be used to generate QALYs [43]. Resource use (dental surgery) will be obtained from the BSA. The participant questionnaire administered at baseline, 6, 12 and 18 months will include healthcare used due to problems with teeth, mouth or dentures, out-of-pocket expenses (e.g. travel, prescriptions costs of antibiotics and analgesics and over the counter medicines) and absence from paid employment. Productivity costs (time away from work) will use human capital-based estimates [38]. Medication cost will be obtained from the British National Formulary and the Drug Tariff. The currency used will be the pound sterling (£).

The within trial analyses aim to assess the incremental cost per QALY gained and will present an incremental cost-effectiveness ratio (ICER). The nonparametric bootstrapping approach will be used to determine the level of sampling uncertainty around the ICER. Cost Effectiveness Acceptability Curves will be presented and net monetary benefit will be calculated. Sensitivity analyses will include estimation of the cost effectiveness using the trial co-primary outcomes (OHIP-14) and attendance at a dental practice for a planned care appointment within 12 months.

### Oversight and monitoring

#### Composition of the coordinating centre and trial steering committee {5d}

A Programme Steering Committee (PSC) including an independent chair, independent psychologist, biostatistician and public involvement advisor will monitor the trial against milestones and advise on methodological aspects and implementation issues. Given that adverse effects of the intervention are judged to be limited, the PSC have identified that an Independent Data and Safety Monitoring Committee is not required. Instead, safety will be reviewed by the PSC. The PSC will meet biannually or as required. A Trial Management Group (TMG) comprising the Chief Investigator (CI), other lead investigators involved in the trial (clinical and non-clinical) and members of the LCTC will meet at least monthly or as required and will be responsible for monitoring trial progress and conduct of the trial.

#### Composition of the data monitoring committee, its role and reporting structure {21a}

No data monitoring committee will be involved initially because the intervention has no major safety concerns. If the PSC feel that unblinded data monitoring is required at any point, then this decision may be revisited.

#### Adverse event reporting and harms {22}

An adverse event (AE) is classified as one occurring between randomisation and the final follow-up contact at 18 months and is any untoward occurrence which does not necessarily have a causal relationship with the RETURN intervention. Serious adverse events (SAE) are AE which results in death, is life threatening, or is life changing. A related AE is that which is deemed to be a result from administration of research procedures. Due to the type of intervention, SAEs and related adverse events are not expected. However, it is possible that administration of the intervention may lead to increased dental anxiety. Modified Dental Anxiety Scale (MDAS) [35] scores are collected at baseline, 6, 12 and 18 months. MDAS items are summed together to produce a total score ranging from 5 to 25 with higher scores relating to higher levels of dental anxiety [35]. If there is a shift from an MDAS quintile of 0-5/6 (low dental anxiety) at baseline to 19-25 (very high) at 6, 12 or 18 months, this will be reported as an AE. At each site there will be a designated investigator named on the ‘signature list and delegation of responsibilities log’ who is responsible for reporting SAEs and making decisions as to whether this constitutes a SAE and whether this is related or unrelated to trial conduct or intervention, and whether unexpected or not.

#### Frequency and plans for auditing trial conduct {23}

Data will be entered into a validated database and during data processing there will be checks for missing or unusual values (range checks) and for consistency within participants over time. Other data checks relevant to patient rights and safety will also be regularly performed as per LCTC processes. Any suspect data will be returned to the site in the form of data queries. Data query forms will be produced at the LCTC from the trial database and sent either electronically or through the post to a RETURN trained staff member (as listed on the site delegation log). RETURN trained staff members will respond to the queries, providing an explanation/resolution to the discrepancies and return the data query forms to the LCTC.

Site monitoring visits may be ‘triggered’ in response to concerns regarding trial conduct, participant recruitment, outlier data or other factors as appropriate. In order to perform their role effectively, the Trial Coordinator and researchers involved in Quality Assurance and Inspection may need direct access to primary data, e.g. patient medical records, appointment books. Since this affects the participant’s confidentiality, this fact is included on the patient information sheet. In agreeing to participate in this trial, a PI grants permission to the Sponsor (or designee), to conduct on-site monitoring and/or auditing of all appropriate trial documentation. The LCTC performs all clinical trial monitoring on behalf of sponsor and if therefore not independent.

#### Plans for communicating important protocol amendments to relevant parties (e.g. trial participants, ethical committees) {25}

All protocol amendments will only be made with the approval of the sponsor and REC. A study website www.returnproject.co.uk will provide updates of any significant changes.

### Dissemination plans {31a}

A full report will be provided to the funded and published by NIHR. There will be a programme of dissemination at the end of the study which includes presentation to health services, policy and lay audiences. A study website is available www.returnproject.co.uk.

## Discussion

This trial is at the pragmatic end of the spectrum of clinical trial design, with its purpose focused on evaluating the effectiveness of the intervention under the usual condition in which it might be applied, whilst maintaining internal validity [44]. This presents particular challenges to its implementation since dental practice activity sits within an independent contractor model with dental staff remunerated according to Units of Dental Activity undertaken. Dental practitioners are known to perceive financial rewards for being involved in research as relatively unattractive, while also feeling they have relatively little knowledge and skills in the area [45]. Integration of trial processes within routine workloads is a key lesson from previous trials situated in dental practice, and hence a flexibility was built into the study flow diagram so that trial processes and delivery of the intervention could be undertaken at any time during the urgent dental care appointment [46].

Previous trials in dental practice have found significant differences in intervention effects between dental practices [47] and so it will be important to examine differences by site type (dental practice [delivering in-hours urgent care and out of hours care], Dental Hospital), as well as incorporating measures to ensure and monitor fidelity of intervention delivery. The RETURN trial will be supported by embedded qualitative work to support understanding the contextual issues influencing the implementation of the intervention, and the trial itself.

## Trial status

Protocol Version number 4.0 date 08/12/2021. First patient recruited 18.08.21. Approximate date when recruitment completed is 17.08.22.

## Data Availability

As per the data controller agreement, the following organisations which have access to the final dataset are The University of Liverpool and the University of Leeds.

## Abbreviations

AE: Adverse event
A&E: Accident & Emergency department
BSA: Business Services Authority
CI: Chief Investigator
CRF: Case Report Form
DH: Dental Hospital
EQ-5D-5L: Generic quality of life questionnaire
GP: General Practice
GPs: General Practitioners
ICER: Incremental cost-effectiveness ratio
IMD: Index of Multiple Deprivation
LCTC: Liverpool Clinical Trials Centre
MDAS: Modified Dental Anxiety Scale
NHS: National Health Service
NIHR: National Institute for Health and Care Research
OHIP: Oral Health Impact Profile
PI: Principal Investigator
PISC: Participant Information Sheet and Consent form
PSC: Programme Steering Committee
QALY: Quality-adjusted life years
REC: Research Ethics Committee
SAE: Serious adverse event
TMG: Trial Management Group
